# Comparison of visual diagnostic accuracy of dermatologists practicing in Germany in patients with light skin and skin of color

**DOI:** 10.1038/s41598-024-59426-4

**Published:** 2024-04-16

**Authors:** Frederik Krefting, Maurice Moelleken, Stefanie Hölsken, Jan-Malte Placke, Robin Tamara Eisenburger, Lea Jessica Albrecht, Alpaslan Tasdogan, Dirk Schadendorf, Selma Ugurel, Joachim Dissemond, Wiebke Sondermann

**Affiliations:** 1 Department of Dermatology, Venereology and Allergology, University Hospital Essen, University Duisburg-Essen, Hufelandstr. 55, 45122 Essen, Germany; 2 Institute of Medical Psychology and Behavioral Immunobiology, University Hospital Essen, University of Duisburg-Essen, 45122 Essen, Germany; 3 Center of Translational Neuro- and Behavioral Sciences (C-TNBS), University Hospital Essen, University of Duisburg-Essen, 45122 Essen, Germany; 4 German Cancer Consortium, Partner Site Essen, Essen, Germany; 5 Research Alliance Ruhr, Research Center One Health, University Duisburg-Essen, Essen, Germany; 6 National Center for Tumor Diseases (NCT-West), Campus Essen, Essen, Germany

**Keywords:** Diagnosis, Physical examination, Skin diseases

## Abstract

Visual clinical diagnosis of dermatoses in people of color (PoC) is a considerable challenge in daily clinical practice and a potential cause of misdiagnosis in this patient cohort. The study aimed to determine the difference in visual diagnostic skills of dermatologists practicing in Germany in patients with light skin (Ls) and patients with skin of color (SoC) to identify a potential need for further education. From April to June 2023, German dermatologists were invited to complete an online survey with 24 patient photographs depicting 12 skin diseases on both Ls and SoC. The study’s primary outcomes were the number of correctly rated photographs and the participants’ self-assessed certainty about the suspected visual diagnosis in Ls compared to SoC. The final analysis included surveys from a total of 129 dermatologists (47.8% female, mean age: 39.5 years). Participants were significantly more likely to correctly identify skin diseases by visual diagnostics in patients with Ls than in patients with SoC (72.1% vs. 52.8%, *p* ≤ 0.001, OR 2.28). Additionally, they expressed higher confidence in their diagnoses for Ls than for SoC (73.9 vs. 61.7, *p* ≤ 0.001). Therefore, further specialized training seems necessary to improve clinical care of dermatologic patients with SoC.

## Introduction

Visual clinical diagnosis plays a key role in clinical dermatology^1,2^. It is usually the first step in clinical routine and often paves the way for subsequent examinations such as bacteriological swab, the collection of scales, hair or nail material for mycological detection or the performance of skin biopsies for histopathological evaluation^3–7^. Therefore, initial visual misinterpretation of clinical findings can have major implications and delay the adequate treatment.

Visual diagnosis of skin diseases in people of color (PoC) is a major challenge in clinical practice^8–13^ and a potential cause of misdiagnosis in this patient cohort. For instance, Gupta et al*.* surveyed 125 residents in the United States (U.S.) regarding their self-assessed confidence in describing, diagnosing, and treating skin conditions in patients with light skin (Ls) and patients with skin of color (SoC)^11^. Across all items, respondents reported increased uncertainty when examining patients with SoC^11^. Further evidence is presented from another survey of dermatology residents in the U.S., according to which 91% of respondents supported additional training on skin conditions in patients with SoC^12^. In line with this observation, medical students in the U.S. misjudged skin diseases more frequently in PoC than in patients with Ls in a study of Fenton et al*.*^8^.

One reason for this difference might be the underrepresentation of skin diseases on SoC in classical dermatology textbooks. An analysis of commonly used textbooks in the U.S. in 2021 showed that only 4–19% of the illustrations were cases of PoC^13^. This lack of diversity in dermatology textbooks has also been described in Germany^14^. In the dermatology textbook “Braun-Falco’s Dermatology, Venereology, and Allergology,” which is a commonly known textbook in German-speaking countries, only approximately 4% of the illustrations showed PoC with skin conditions^14,15^. In the textbook “Dermatologie Venerologie: Grundlagen, Klinik, Atlas” published by Fritsch and Schwarz in 2018, as little as 0.54% of all images present patients with SoC^14,16^. A representative number of skin diseases on SoC can only be found in textbooks that focus on this topic^17,18^.

To the best of our knowledge, studies or surveys on the medical care of skin diseased PoC, as already conducted in the U.S. and described above, have rarely been published in Europe^8,11,12^. In particular, there is a lack of studies focusing on the visual diagnostic accuracy and certainty of dermatologists in Germany for patients with Ls and patients with SoC. In order to fill this gap, we conducted a nation-wide online survey among dermatologists practicing in Germany, who assessed the visual diagnostic skills of physicians in skin-diseased patients with Ls and patients with SoC to evaluate the need for training.

## Results

A total of N = 129 completely (65.8%, 85/129) or partially (34.2%, 44/129) filled surveys were included in the analysis. Almost half of the participants were female (47.8%, 61/129), and the median age of the cohort was 39.5 years. Most respondents with complete known demographic information had a board certification in dermatology (63.5%, 54/85), practiced in a clinic (74.1%, 63/85), and identified as Caucasians (98.8%, 84/85). Respondents estimated to see a median of 5.2% of patients with SoC in their clinical practice. Participation in special courses for the treatment of patients with SoC was reported only in some rare cases (8.2%, 7/85).

Participants were significantly more likely to correctly identify skin diseases by visual diagnostics on patients with Ls than on patients with SoC (72.1% vs. 52.8%, *p* ≤ 0.001, OR 2.28).

The comparison of the individual diseases showed that participants were significantly more likely to correctly identify the diseases tinea corporis, psoriasis, melanonychia, herpes zoster, atopic dermatitis, toxic epidermal necrolysis (TEN), herpes simplex, and acne vulgaris in patients with Ls than in patients with SoC.

In contrast, participants were significantly better at detecting tinea capitits in PoC than in patients with Ls. Acne keloidalis nuchae was also more frequently correctly diagnosed in patients with SoC than in patients with Ls, however, this difference was not statistically significant. Photographs of folliculitis decalvans and Kaposi’s sarcoma were correctly identified with a similar probability in both groups.

**Figure 1:**
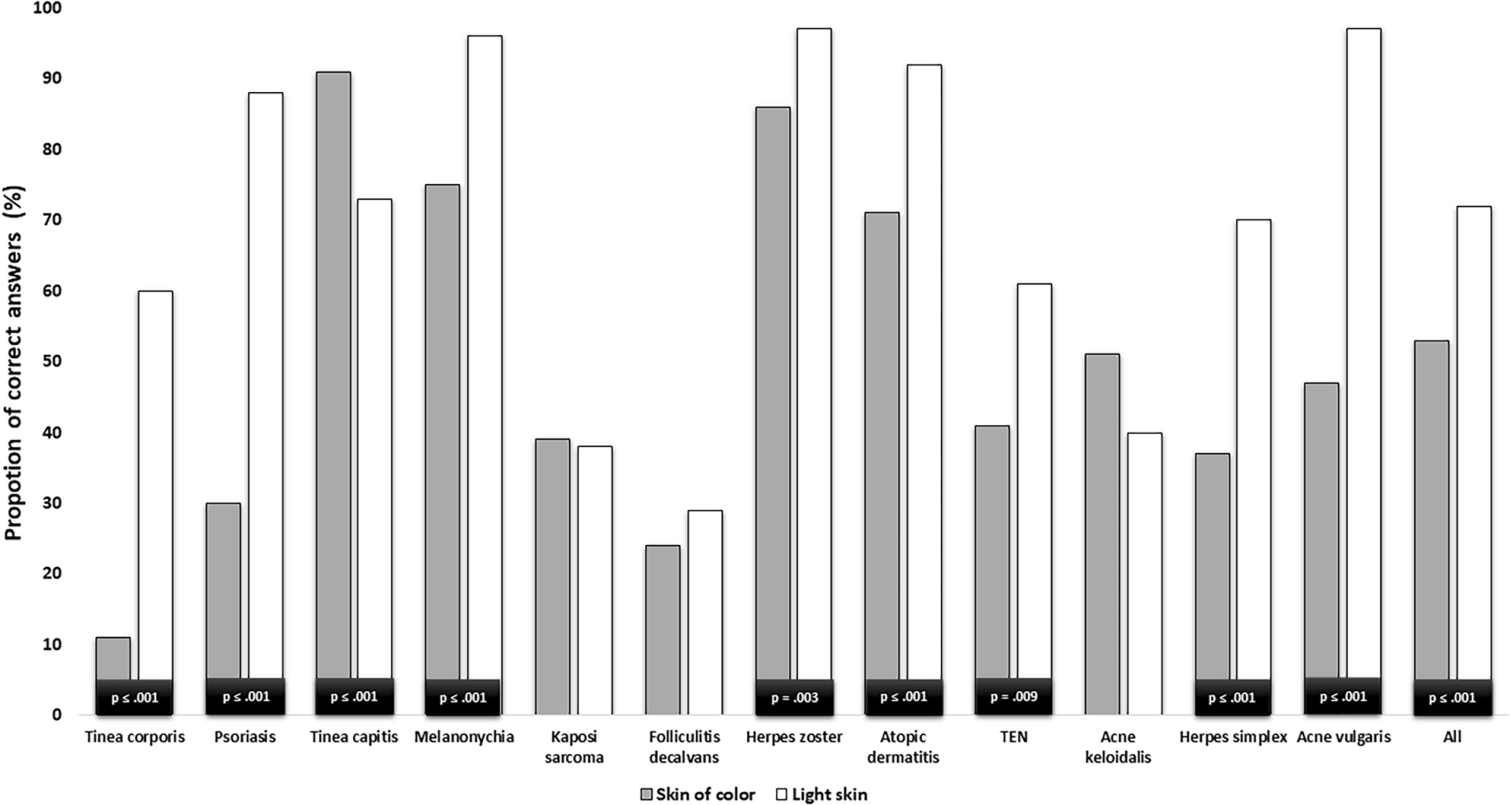
Comparison of correct diagnoses between the skin types. Abbreviations: TEN = Toxic epidermal necrolysis, Acne keloidalis = Acne keloidalis nuchae. *p*-values are shown for statistically significant chi-square-tests at α = 0.05.

The results of the described comparison of visual diagnostic accuracy including the respective statistical information are shown in Fig. 1 and summarized in Table 1 (Fig. 1, Table 1).

**Table 1:** Comparative results of diagnostic accuracy.

Investigated dermatoses																										
Items	Tinea corporis		Psoriasis		Tinea capitis		Melano-nychia		Kaposi sarcoma		Folliculitis decalvans		Herpes zoster		Atopic dermatitis		TEN		Acne keloidalis		Herpes simplex		Acne vulgaris		All	
Analysis	Relation of correct answers																									
Skin type	Ls	SoC	Ls	SoC	Ls	SoC	Ls	SoC	Ls	SoC	Ls	SoC	Ls	SoC	Ls	SoC	Ls	SoC	Ls	SoC	Ls	SoC	Ls	SoC	Ls	SoC
Numbers of all analyzed answers	90	96	89	89	90	97	90	98	93	89	94	101	94	95	91	91	86	86	91	94	86	87	95	93	1189	1116
Relation of correct answers (%)	60.0	11.5	88.8	30.3	73.3	91.8	96.7	75.5	38.7	39.3	29,7	24,5	97.9	86.3	92,3	71,4	61.6	41.9	40.7	51.1	70.9	37.9	96.8	46.7	72.1	52.8
*p*-value, chi-square-test	< 0.001		< 0.001		< 0.001		< 0.001		0.932		0.412		0.003		< 0.001		0.009		0.156		< 0.001		< 0.001		< 0.001	
Odds ratio [95% CI]	11.59 [5.44–2.49]		18.14 [8.16–40.29]		0.24 [.10-.58]		9.40 [2.72–32.48]		0.97 [.53–1.76]		1.30 [.69–2.46]		7.29 [1.59–33.28]		4.80 [1.96–11.74]		2.23 [1.21–4.10]		0.65 [.36–1.17]		3.99 [2.11–7.54]		34.94 [10.31–118.44]c		2.28 [1.92–2.71]	

*SoC* skin of color, *Ls* light skin, *95% CI* 95% confidence interval.

Further subgroup analyses showed that board certified dermatologists were significantly more likely to find the correct diagnoses than residents in patients with Ls (76.4% vs. 64.0%, *p* = 0.018) as well as in patients with SoC (55.0% vs. 47.2%, *p* ≤ 0.001). In addition, respondents who had already attended a specialized course on treating patients with SoC were more likely to give the correct diagnosis in PoC. Table 2 summarizes the results of the subgroup analyses (Table 2).

**Table 2:** Comparative results of subgroup analyses.

Investigated dermatoses								
Items	Participants without board certification in dermatology		Participants with board-certification in dermatology		Participants who participated in specific courses for treatment of PoC		Participants who did not participate in specific courses for treatment of PoC	
Skin type	Ls	SoC	Ls	SoC	Ls	SoC	Ls	SoC
Numbers of all analyzed answers	372	362	627	627	23	35	259	438
Relation of correct answers (%)	64.0	47.2	76.4	55.0	70.9	55.7	71.8	51.9
*p*-value, chi-square-test, SoC	0.018				0.514			
*p*-value, chi-square-test, Ls	< 0.001				0.855			

*SoC* skin of color, *Ls* light skin.

The analysis of participants’ self-assessed certainty about the suspected diagnoses showed higher mean scores, i.e., higher certainty, for the diseases on Ls than on SoC (73.9 vs. 61.7, *p* ≤ 0.001).

Consistently, respondents indicated higher diagnostic certainty in patients with Ls for the diseases tinea corporis, psoriasis, Kaposi sarcoma, melanonychia, folliculitis decalvans, herpes zoster, atopic dermatitis, TEN, herpes simplex and acne vulgaris than in those with SoC.

In contrast, evaluation of the photographs of tinea capitis and acne keloidalis nuchae led to higher mean certainty scale scores for the images of PoC than for the patients with Ls.

**Figure 2:**
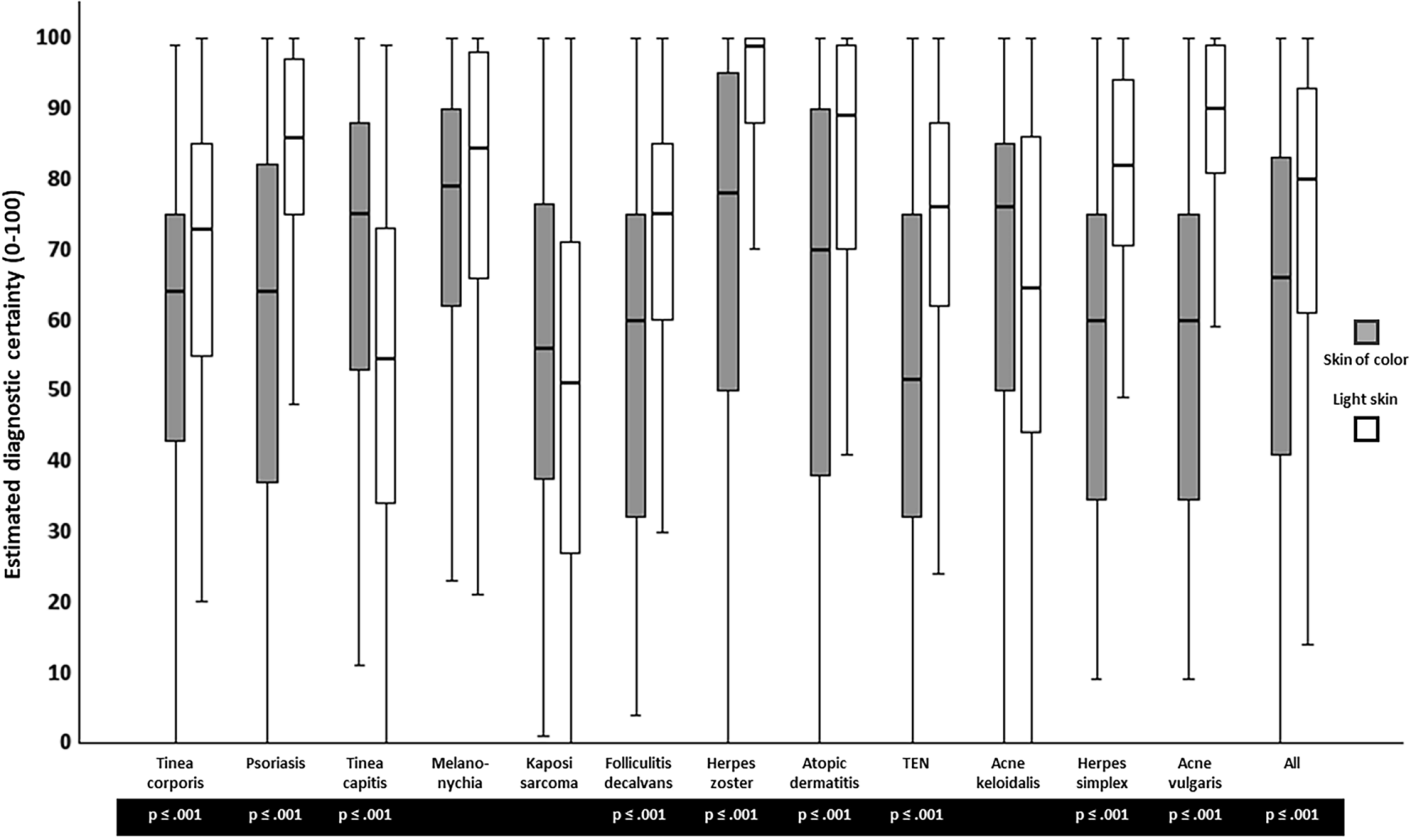
Comparison of self-assessed diagnostic certainty between the skin types using box plot analysis. Rating was given on a visual analogue scale form 0 (completely unsure) to 100 (completely sure). Box plots display median values as centre line, the upper and lower quartiles as bounds of boxes, and the minimum and maximum values as whiskers. Abbreviations: TEN = Toxic epidermal necrolysis, Acne keloidalis = Acne keloidalis nuchae. *p*-values are shown for statistically significant t-test with α = 0.05.

The results of participants’ self-assessed certainty about the suspected diagnoses including the respective statistical information are shown in Fig. 2 and summarized in Table 3 (Fig. 2, Table 3).

**Table 3:** Comparative results of diagnostic certainty.

Investigated dermatoses																										
Items	Tinea corporis		Psoriasis		Tinea capitis		Melano-nychia		Kaposi sarcoma		Folliculitis decalvans		Herpes zoster		Atopic dermatitis		TEN		Acne keloidalis		Herpes simplex		Acne vulgaris		All diseases	
Skin type	Ls	SoC	Ls	SoC	Ls	SoC	Ls	SoC	Ls	SoC	Ls	SoC	Ls	SoC	Ls	SoC	Ls	SoC	Ls	SoC	Ls	SoC	Ls	SoC	Ls	SoC
Analyzed answers (n)	90	90	89	89	91	91	90	90	89	89	91	91	88	88	87	87	90	90	89	89	90	90	90	90	1074	1074
Mean value, diagnostic certainty (0–100)	70.7	58.1	80.2	60.0	52.2	70.1	76.6	74.7	54.2	50.0	72.8	54.2	91.4	67.8	83.2	61.7	72.9	52.3	62.3	67.2	78.1	57.2	85.6	55.7	73.9	61.7
Median value, diagnostic certainty (0–100)	73	64	85	64	54	75	84	79	51	56	75	60	98	78	89	70	75	71	64	76	81	760	89	57	79	66
*p*-value, t-test, mean value	< 0.001		< 0.001		< 0.001		0.431		0.255		< 0.001		< 0.001		< 0.001		< 0.001		0.146		< 0.001		< 0.001		< 0.001	

*SoC* skin of color, *Ls* light skin.

## Discussion

The main aim of this study was to identify possible differences in the skills and confidence of dermatologists practicing in Germany in the visual clinical diagnosis of patients with Ls and patients with SoC in order to identify a particular need for further training. We detected a significant difference in the quality of participants’ visual diagnostic skills between skin types: both the rate of correct suspected diagnoses as well as the subjective self-assessed certainty about the diagnoses were significantly lower for cases of patients with SoC.

In particular, the participants’ correct diagnoses of dermatoses with inflammatory reactions in patients with Ls significantly outweighed the correct diagnoses in patients with SoC. A likely explanation could be the fact that inflammatory dermatoses such as tinea corporis, psoriasis, herpes zoster, atopic dermatitis, TEN, herpes simplex, and acne vulgaris are usually associated with erythema on Ls^19–28^ which provides an important sign for dermatologists to the correct visual diagnosis. On SoC, erythema can be completely missing or appear in violaceous, ashen gray or darker brown color^29–44^. In line with this observation, respondents' self-assessed certainty for these diseases showed lower values for PoC, indicating that respondents were aware of their own uncertainty in treating PoC suffering from these diseases.

The largest difference in diagnostic accuracy between the skin types was observed for tinea corporis. Visual diagnosis of tinea corporis in PoC is a major challenge due to the above-described problem of a lack of characteristic erythema as well as a lack of or poorer demarcation of peripheral scales on SoC than on Ls, so that differential diagnoses such as atopic eczema, impetigo contagiosa, seborrheic dermatitis, psoriasis, systemic lupus erythematosus, or granuloma annulare can be poorly differentiated by visual diagnostics^19,20,32^. Nevertheless, the participants’ rate of only 11% of correct diagnoses of tinea corporis in PoC is worrisome. Of equal concern was the fact that 68.1% of the cohort did not recognize the life-threatening disease of TEN in PoC^28,36,37^. However, not all cases were better and more confidently diagnosed in patients with Ls than in patients with SoC. Tinea capitis and acne keloidalis nuchae were more likely to be detected on SoC than on Ls, and participants also reported a greater certainty on SoC than on Ls. A possible reason explaining these results could be data showing a higher prevalence for these diseases in PoC, possibly leading to increased expectations among participants for these dermatoses in PoC. It is important to note that these conditions can of course affect anyone, regardless of the individual skin type^45–47^. In contrast to these interpretations and findings, participants correctly detected melanonychia significantly more often in Ls than in PoC, although melanonychia is more common in PoC^48–50^. This outcome and also all other findings with higher rates of correct diagnoses and certainty for diseases on Ls could be associated with the fact that participating physicians reported to treat much more patients with Ls than with SoC (5.2%) in their daily routine and were therefore much better trained on Ls.

To generate further information about the assessed cohort, subgroup analyses were carried out. These showed that participants with acquired board certification in dermatology were more likely to correctly classify the photographs on Ls as well as on SoC than the participating residents. In view of the fact that skills of visual diagnostics can be improved by training and experience, this finding is not unexpected^51^.

Another subgroup analysis focused on specific courses for dermatological diagnostic and treatment of PoC. The results indicated that just a few participants had attended special classes so far, but those who had done so performed above average in determining the correct diagnoses in PoC. This finding shows that specific courses are useful to improve the skills of dermatologists and, in turn, the medical care of PoC.

The presented work has some limitations. In view of the response rate of around 3%, a selection bias of participants who are especially interested in the topic cannot be ruled out. However, as such a bias would only suggest an even worse performance of dermatologists in the real world, we still regard our findings as valid. Furthermore, finding a correct diagnosis without any other information about the shown cases is a challenging and somewhat artificial task, potentially leading to the partially low rates of correct answers and explaining the poor results regarding folliculitis decalvans and Kaposi sarcoma for both skin types. In addition, even though great care was taken in choosing the pictures with regard to comparability between skin types, they may have had different individual levels of difficulty for clinical assessment, which may have influenced the results.

The presented study is the first of its kind and offers a so far unique insight into the diagnostic accuracy and confidence of German dermatologists in PoC compared to patients with Ls. The results of our study, involving more than 100 participants, highlight the urgent need for further training of dermatologists working in Germany to improve their diagnostic visual skills in PoC.

Given Germany’s highly diverse society, which includes for example more than one million people of African descent^52,53^, the results of our study, which reflect the current situation in Germany, underscore the urgent need for further education on this important topic.

## Materials and methods

The online survey was conducted between April and June 2023. The Ethics committee of the Medical Faculty of the University Duisburg-Essen gave its approval prior to the start of the study (reference number 23-11100-BO) and all research was performed in accordance with relevant guidelines and regulations. The patients in this manuscript have given written informed consent to publication of their case details. Physicians working in a dermatologic clinic or in private practice in Germany were invited to participate via personal contacts and via the newsletter of the German Dermatologic Society (Deutsche Dermatologische Gesellschaft, DDG, N = approximately 3800 members). All participants gave their written informed consent at the beginning of the questionnaire. Participation was voluntary and could be discontinued at any time without the need to give any reason.

The questionnaire consisted of 24 photographs of patients, which had been collected in the context of the clinical routine, showing 12 different skin diseases, once on Ls and once on SoC to allow for comparisons. However, participants were not informed about this design in advance. The order of cases was randomized between participants. Without being given any additional information about the depicted patient, participants were asked to give a suspected diagnosis based on the images. In addition, they were asked to indicate how certain they were about this diagnosis on a scale from 0 (completely uncertain) to 100 (completely certain). To record the participant characteristics, additional demographic questions were asked about age, gender, ethnicity, work experience, worksite and prior experience or training in the treatment of patients with Soc.

can be found in the Supplemental Material (Supp. Fig. 1). The diagnoses of the following cases had been validated by means of comprehensive diagnostics such as skin biopsies, pathogen-specific-PCR or mycological cultures prior to this study: tinea corporis, tinea capitis, Kaposi sarcoma, folliculitis decalvans, herpes simplex and toxic epidermal necrolysis (TEN). The correct answers to the cases psoriasis, melanonychia, herpes zoster, atopic dermatitis, acne keloidalis nuchae, and acne vulgaris were determined by a team of 4 dermatologists by clinical examination.

To measure differences in the number of correctly rated photographs depending on the skin type, chi-square tests with a significance level of α = 0.05 were used and odds ratios (OR) were calculated. To detect statistically significant differences between the diagnostic certainty per skin type, t-tests with α = 0.05 were performed.

The questionnaire was created using the online tool LimeSurvey (LimeSurvey GmbH, Hamburg, Germany). Figures were created with Microsoft PowerPoint (Microsoft, Redmond, Washington, U.S., version 2306) and IBM SPSS Statistics (Statistical Package for Social Science, SPSS Inc., Chicago, version 27). Statistical analyses were conducted with IBM SPSS Statistics.

### Supplementary Information

Supplementary Figures.

Supplementary Information.

## Data Availability

The data that support the findings of this study are available from the corresponding author upon reasonable request.
