# Supplementary material for: Comparison of visual diagnostic accuracy of dermatologists practicing in Germany in patients with light skin and skin of color
Source: Sci Rep. 2024 Apr 16;14:8740. doi: 10.1038/s41598-024-59426-4 (PMC11021442; doi:10.1038/s41598-024-59426-4)
Supplement: Supplementary file 1 — Supplementary Figures. [file 41598_2024_59426_MOESM1_ESM.pdf]

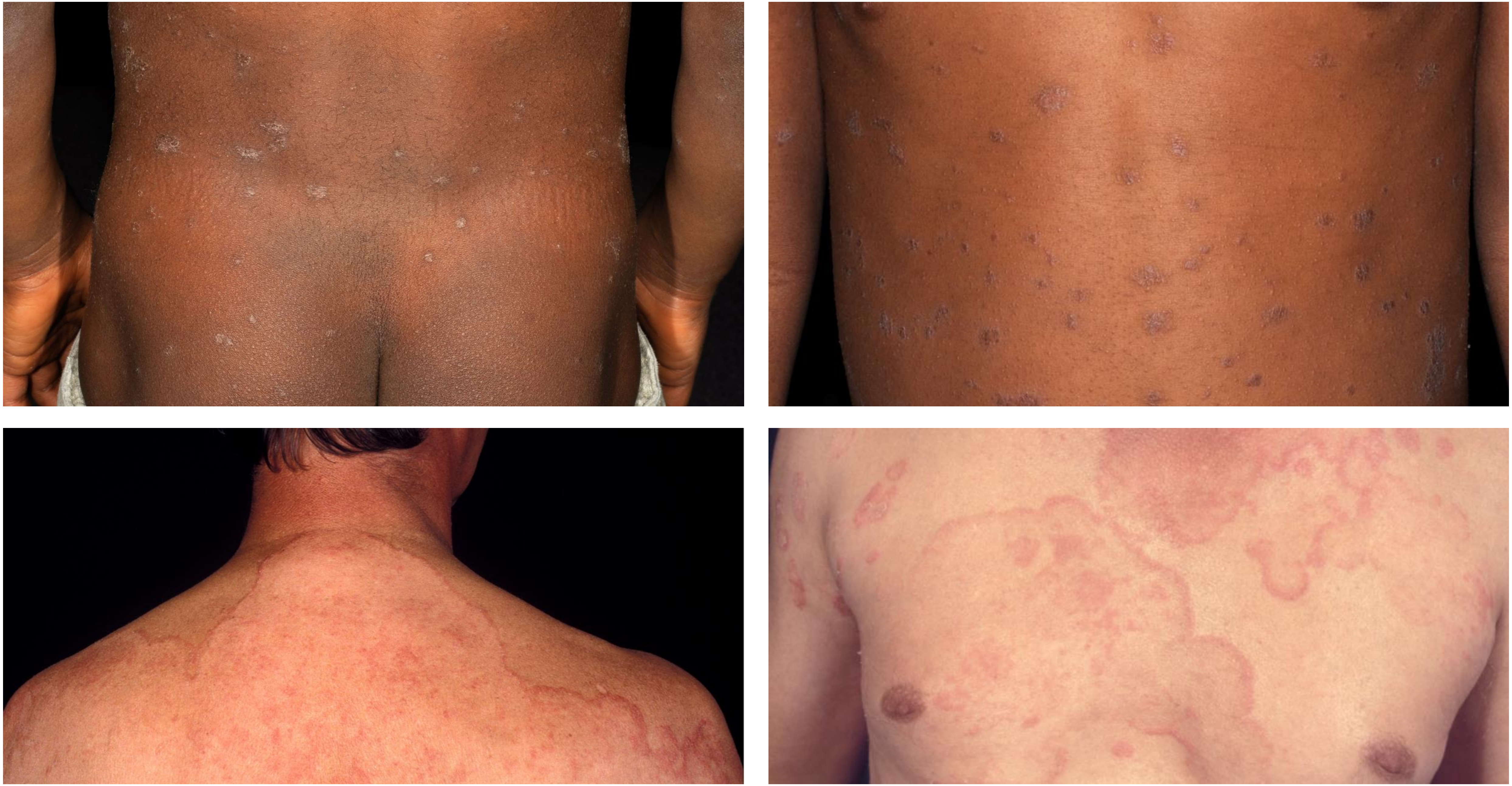

Correct answers: Tinea corporis, mycosis

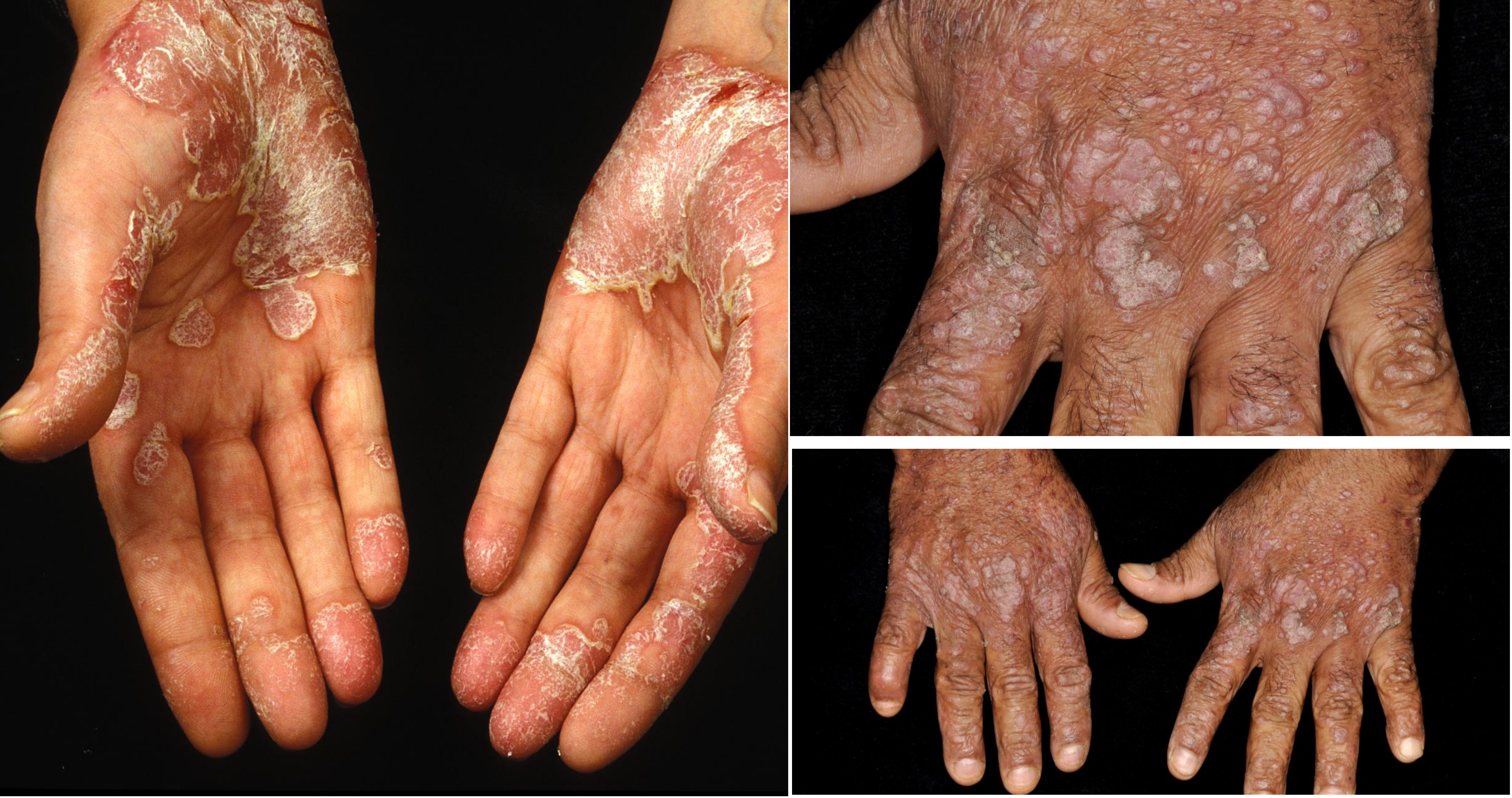

Correct answer: Psoriasis

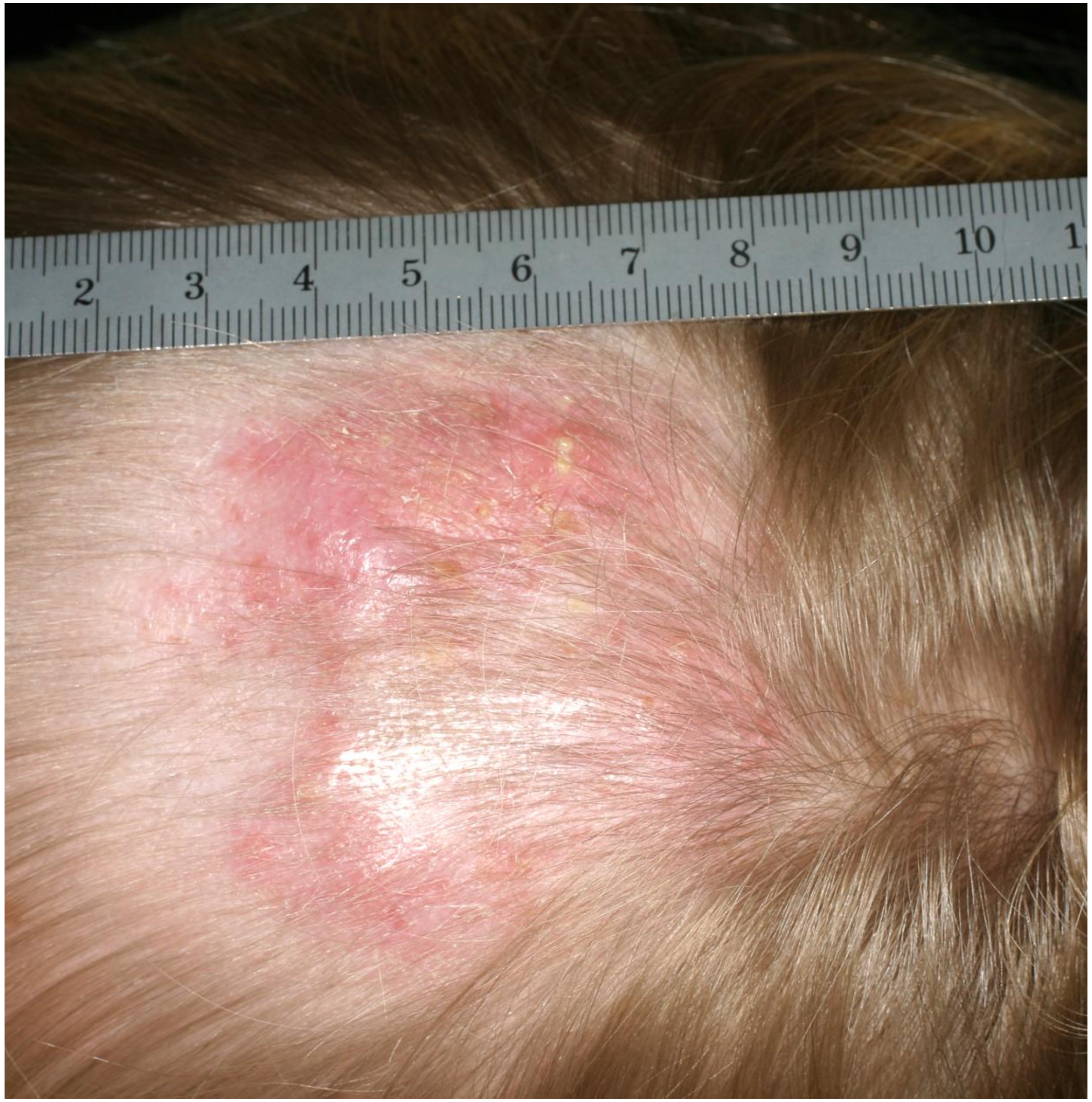

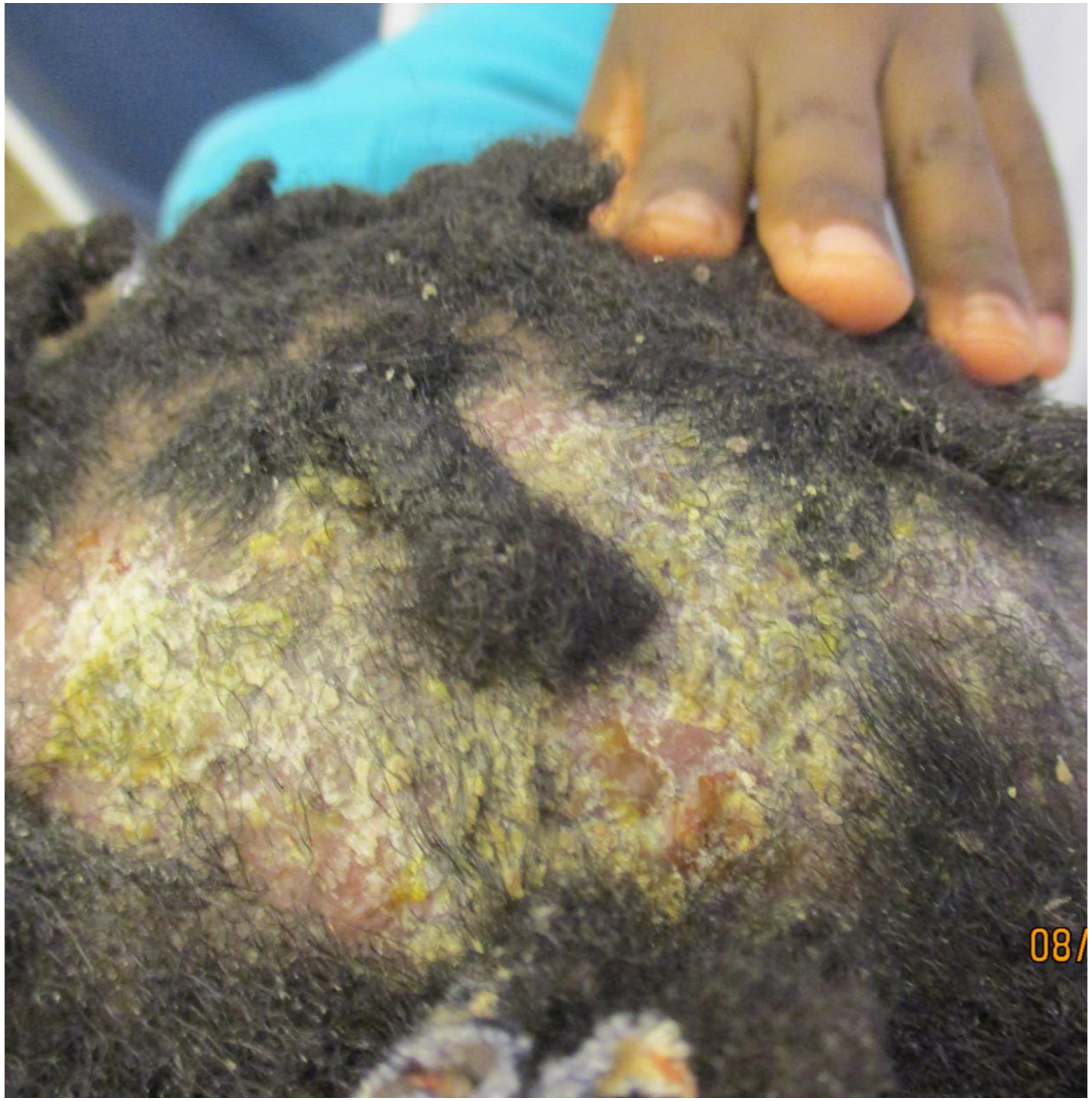

Correct answers: Tinea capitis, kerion celsi, trichophytia, flavus, mycosis

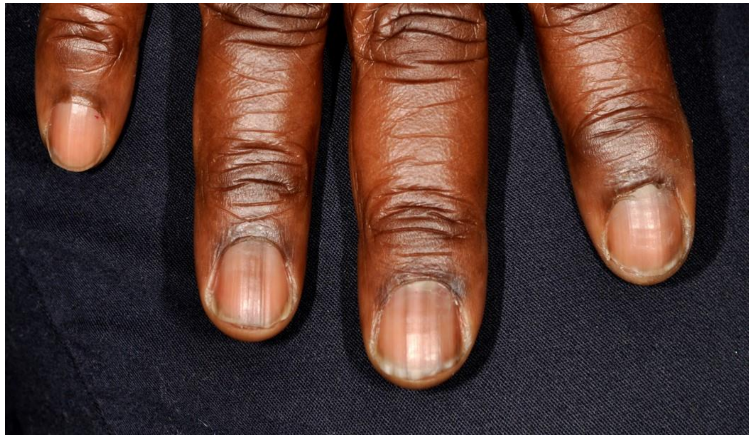

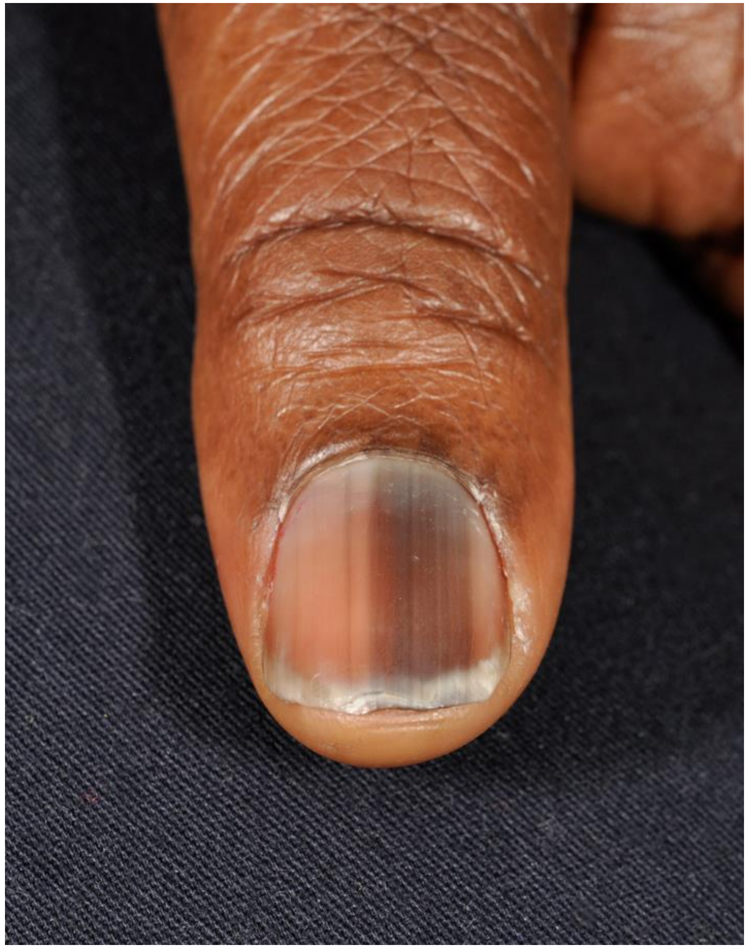

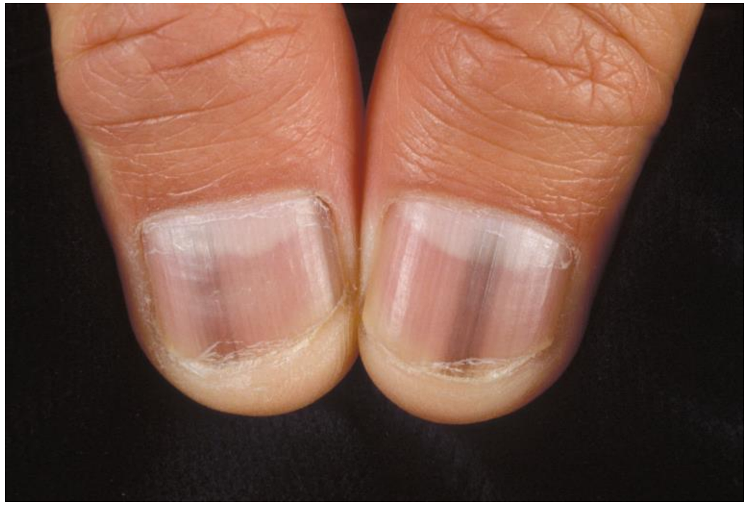

Correct answers: Melanonychia, naevus

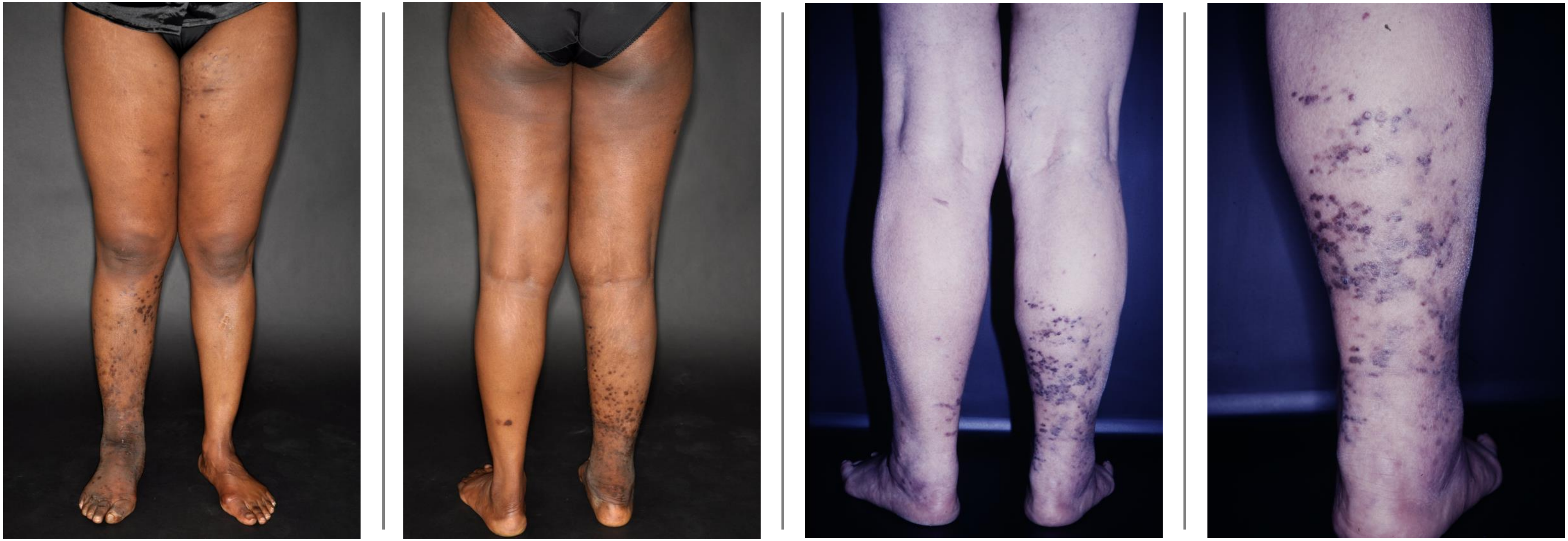

Correct answer: Kaposi sarcoma

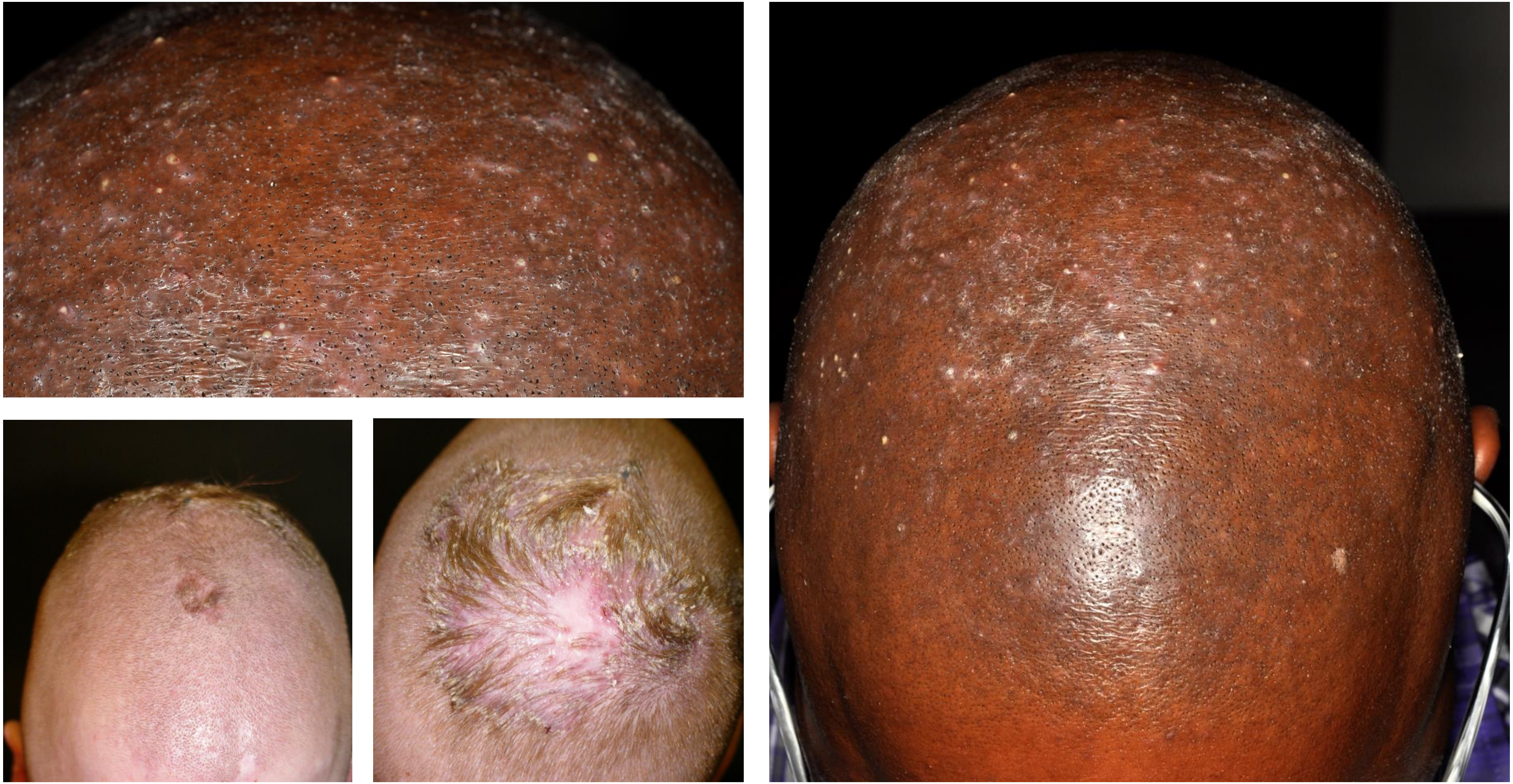

Correct answers: Folliculitis decalvans

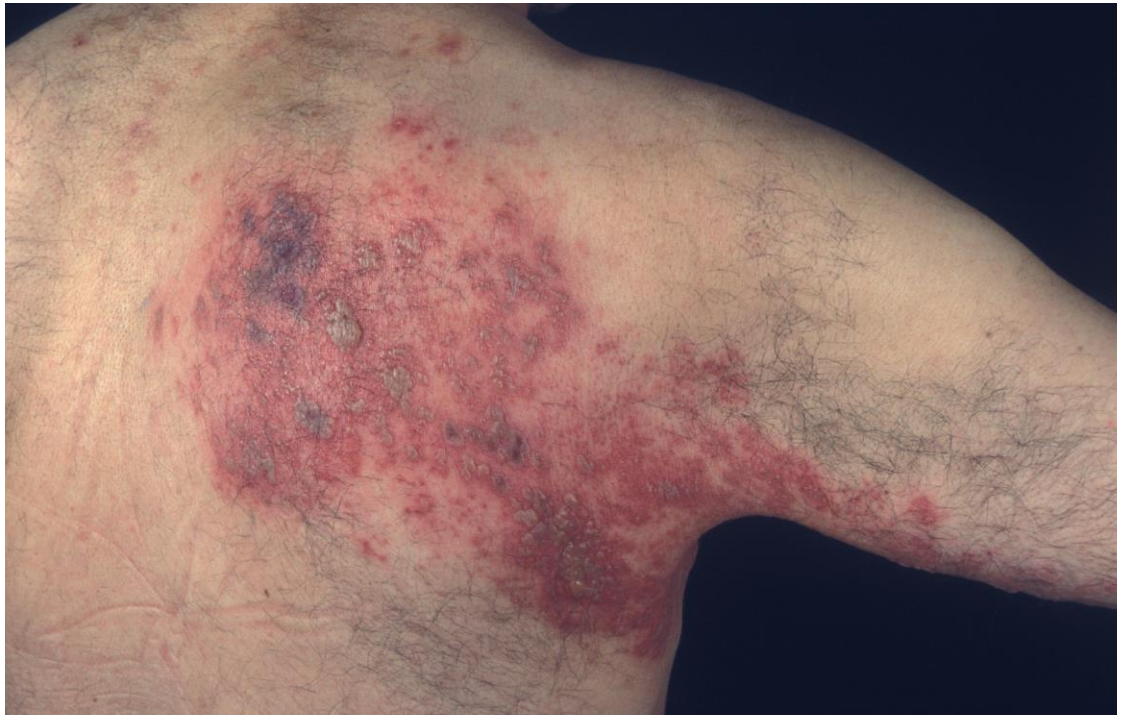

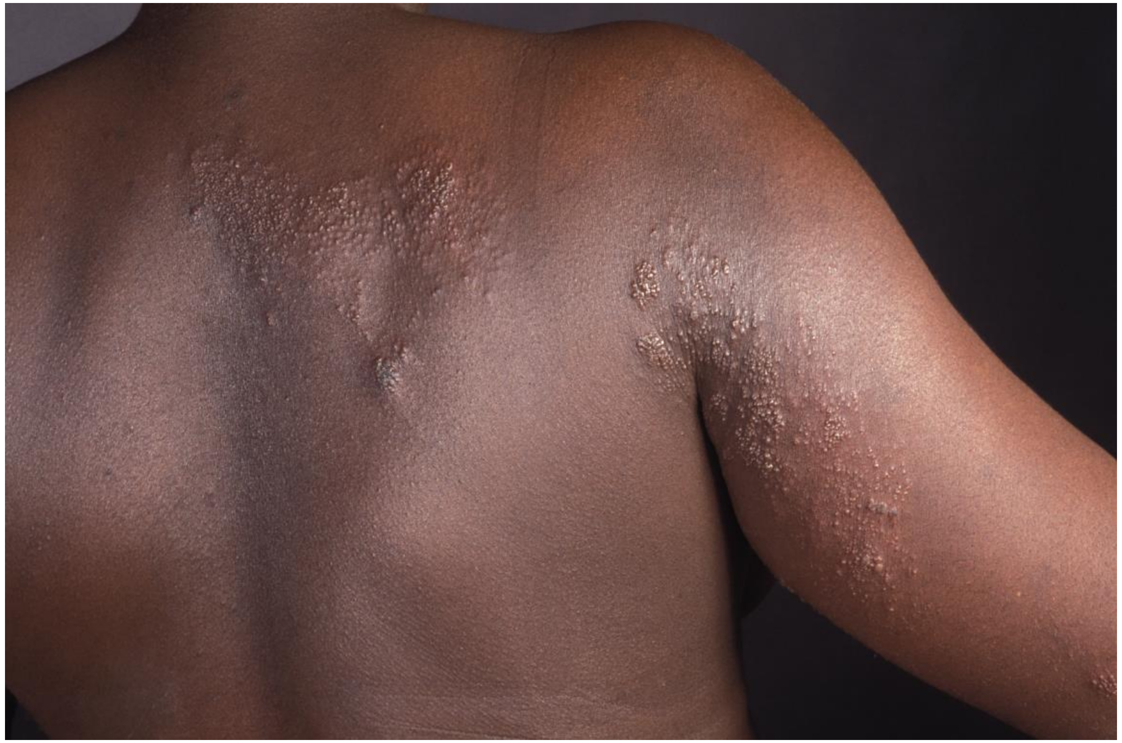

Correct answers: Herpes zoster

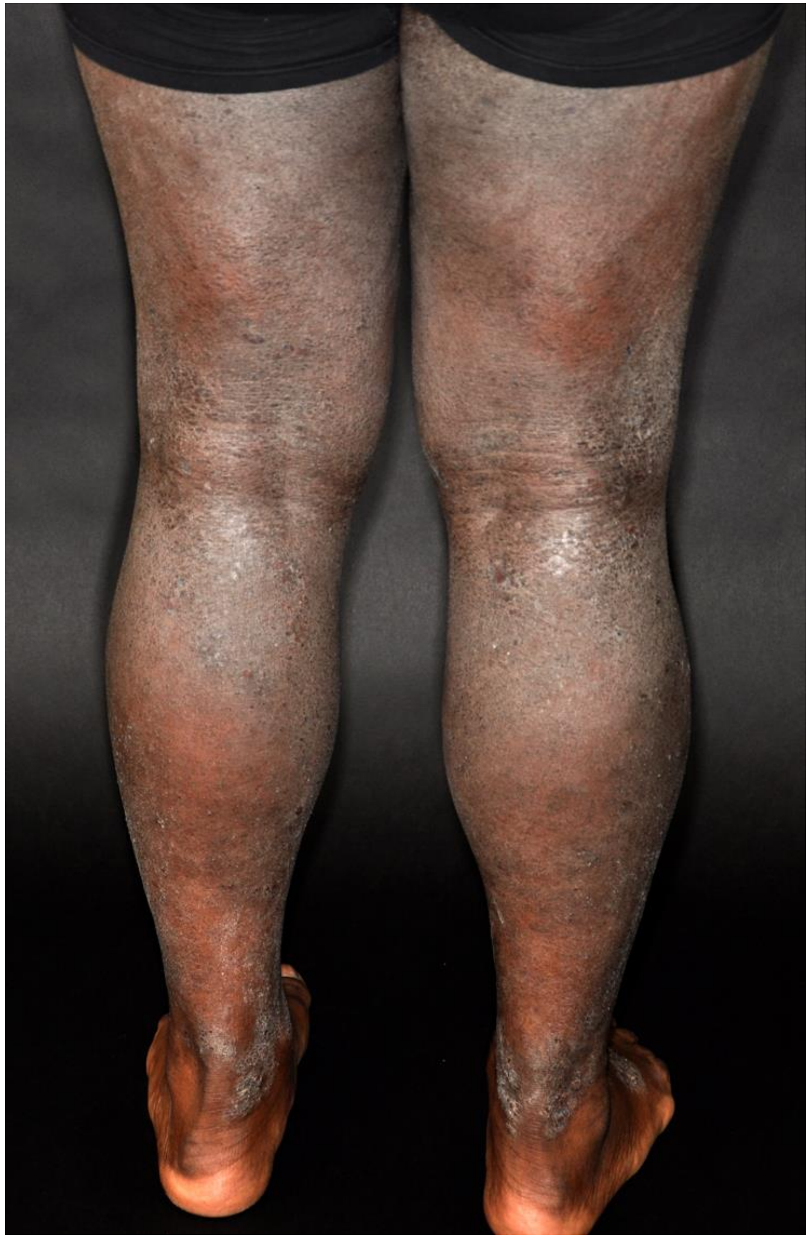

Correct answers: Atopic dermatitis

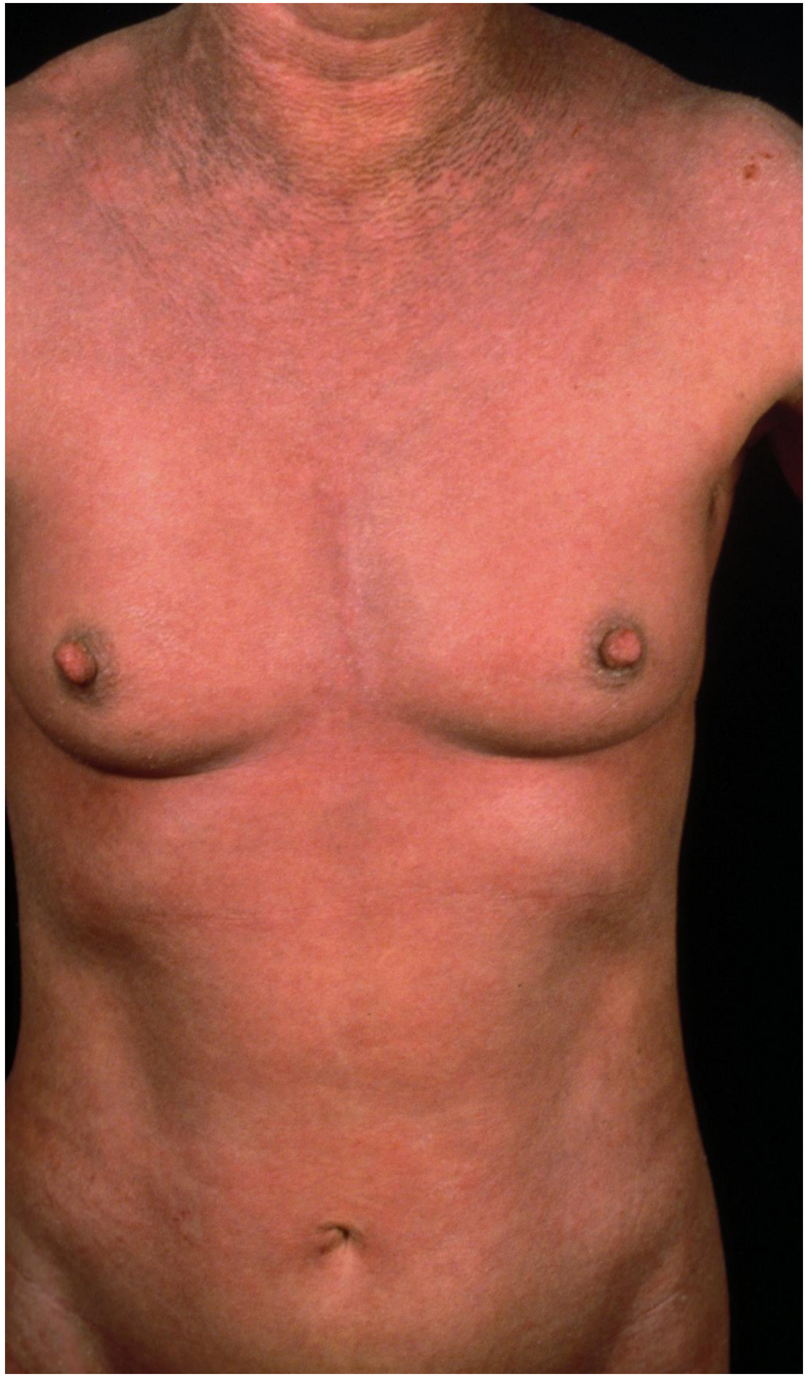

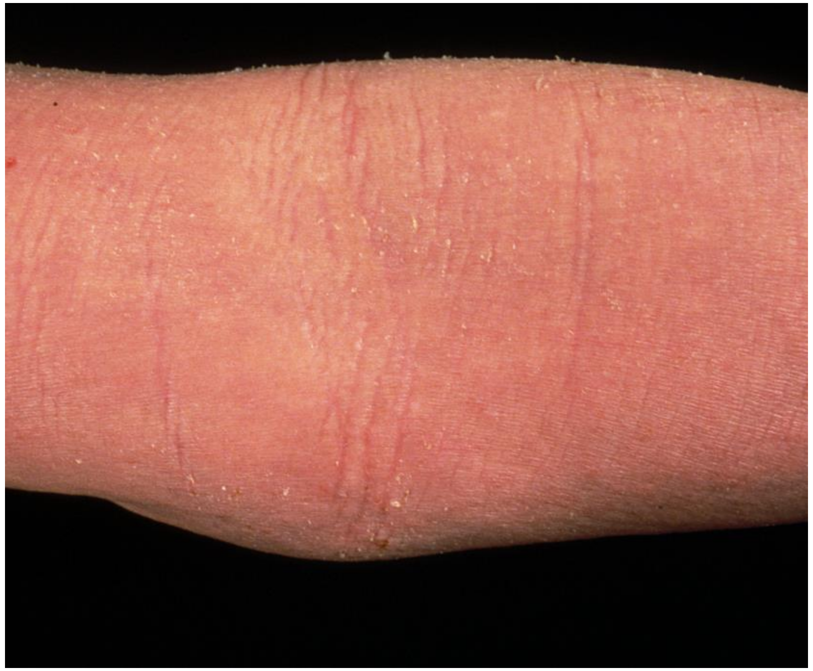

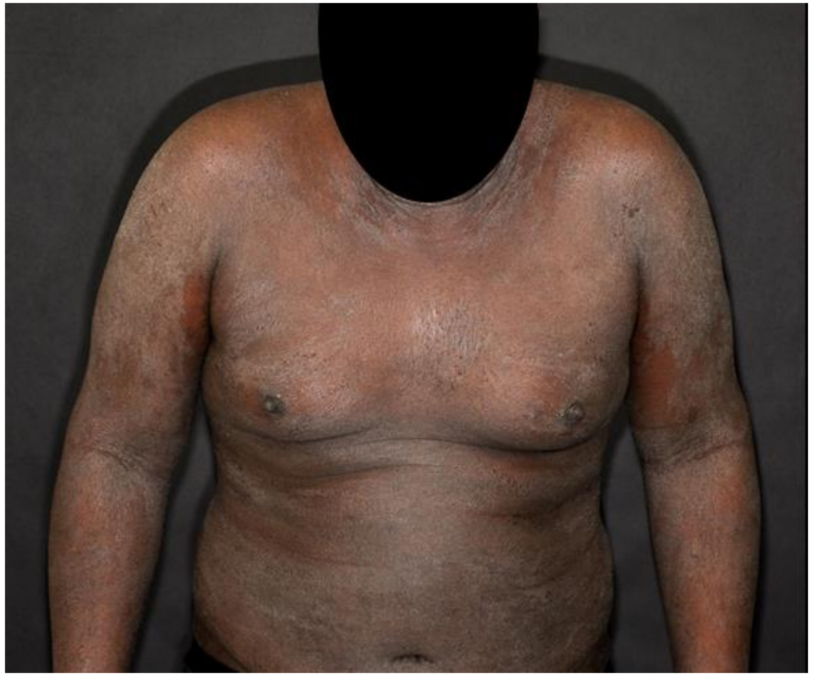

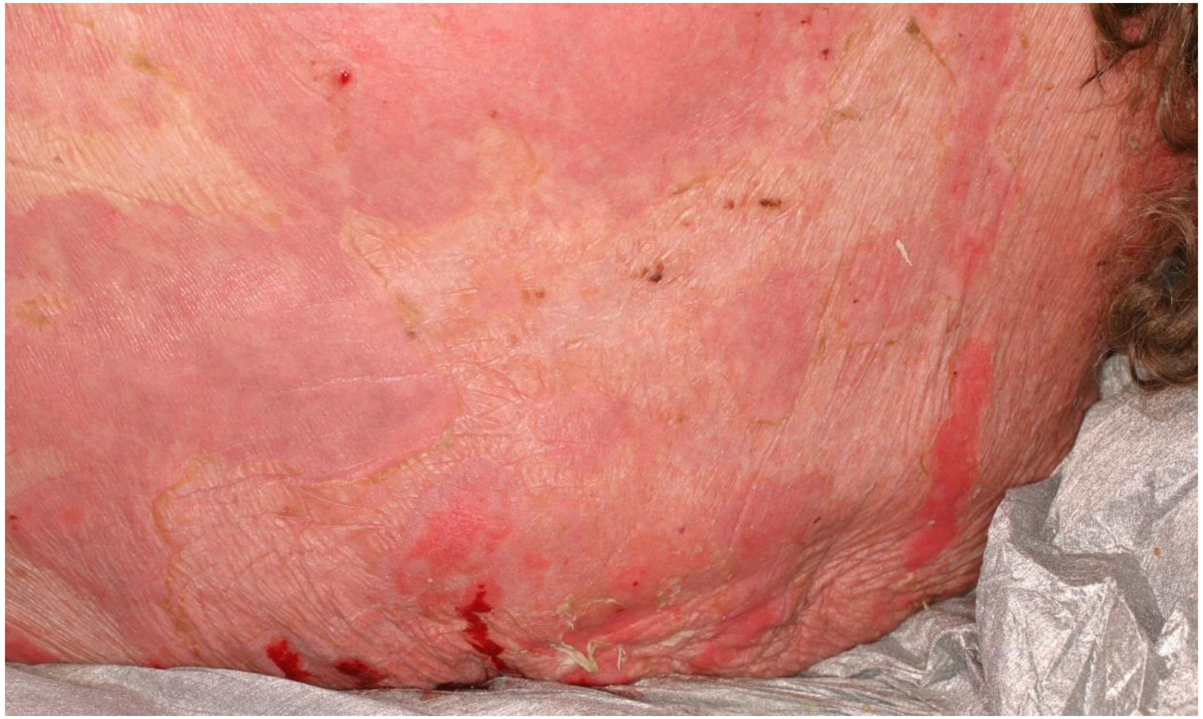

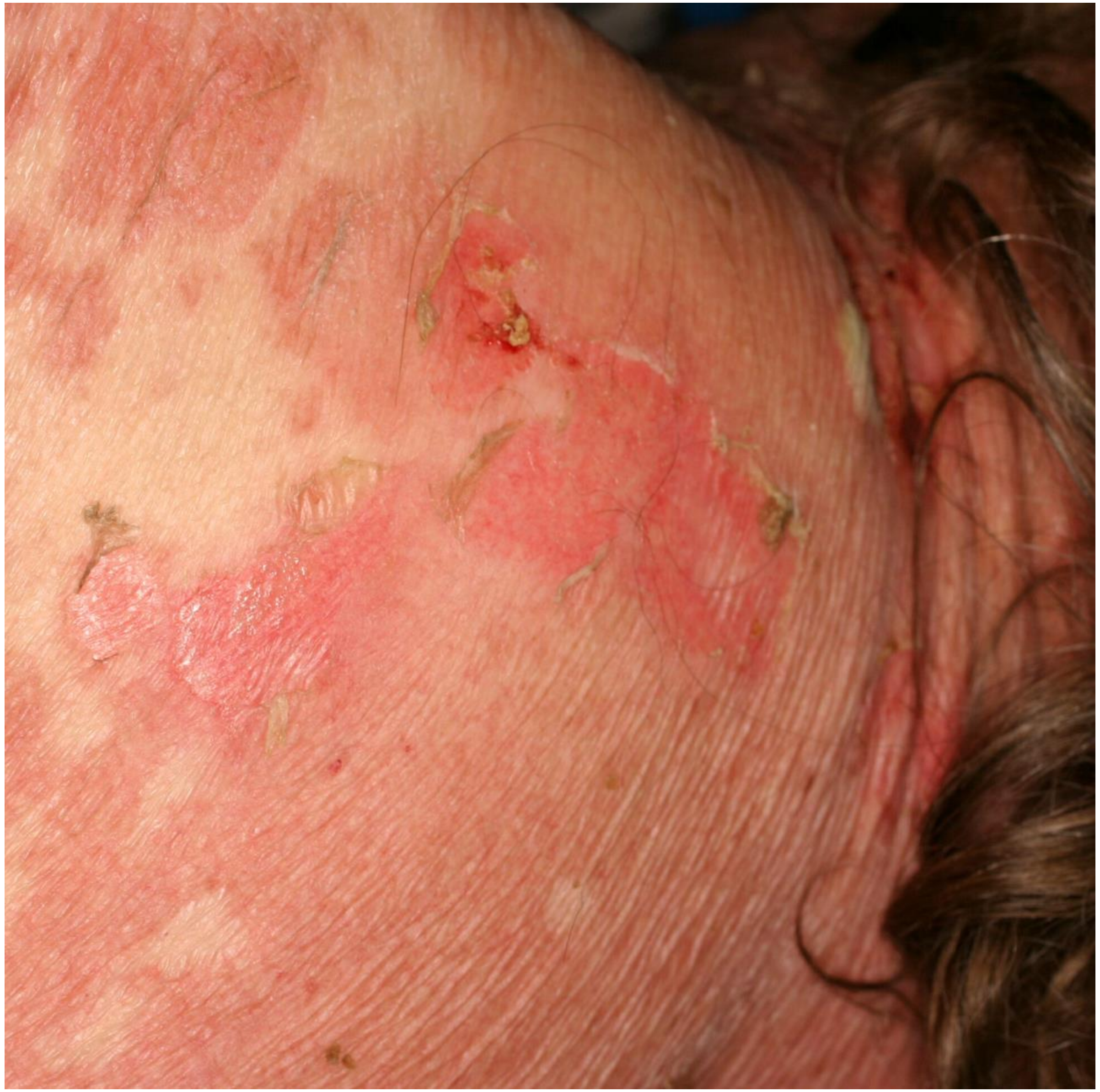

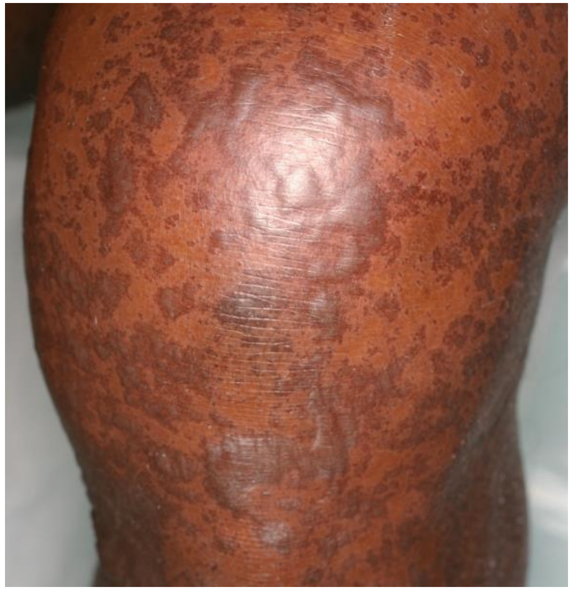

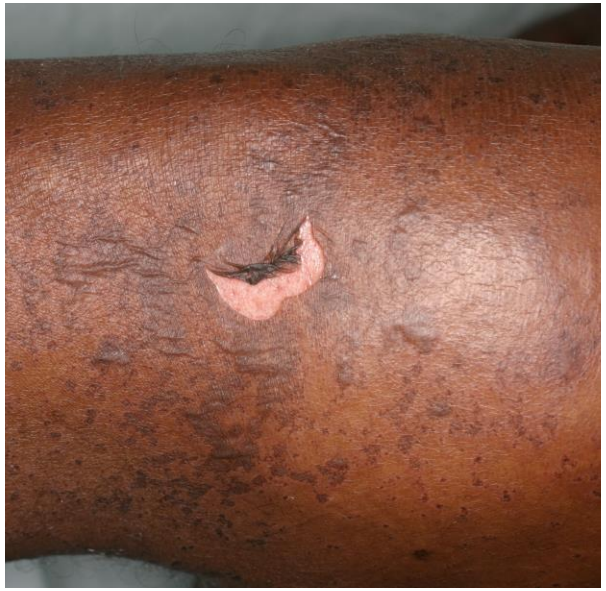

Correct answers: Steven-Johnson-syndrome, toxic epidermal necrolysis

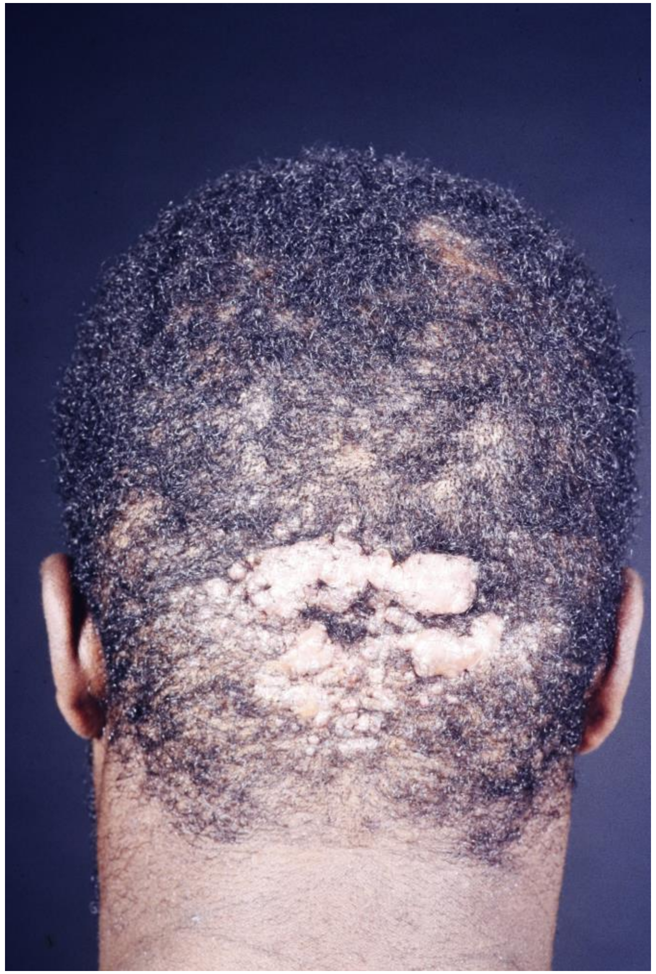

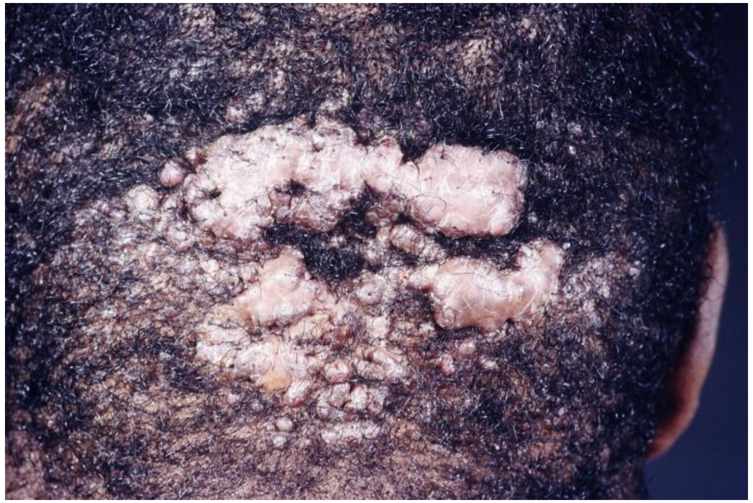

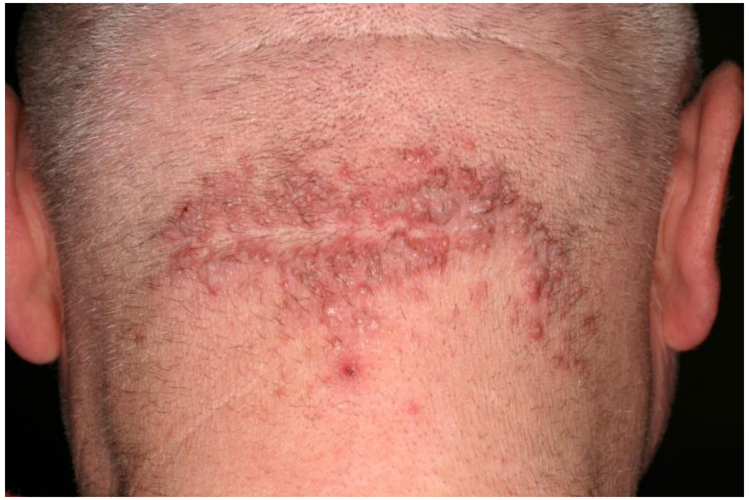

Correct answers: Acne keloidalis nuchae, Folliculitis sclerotisans nuchae

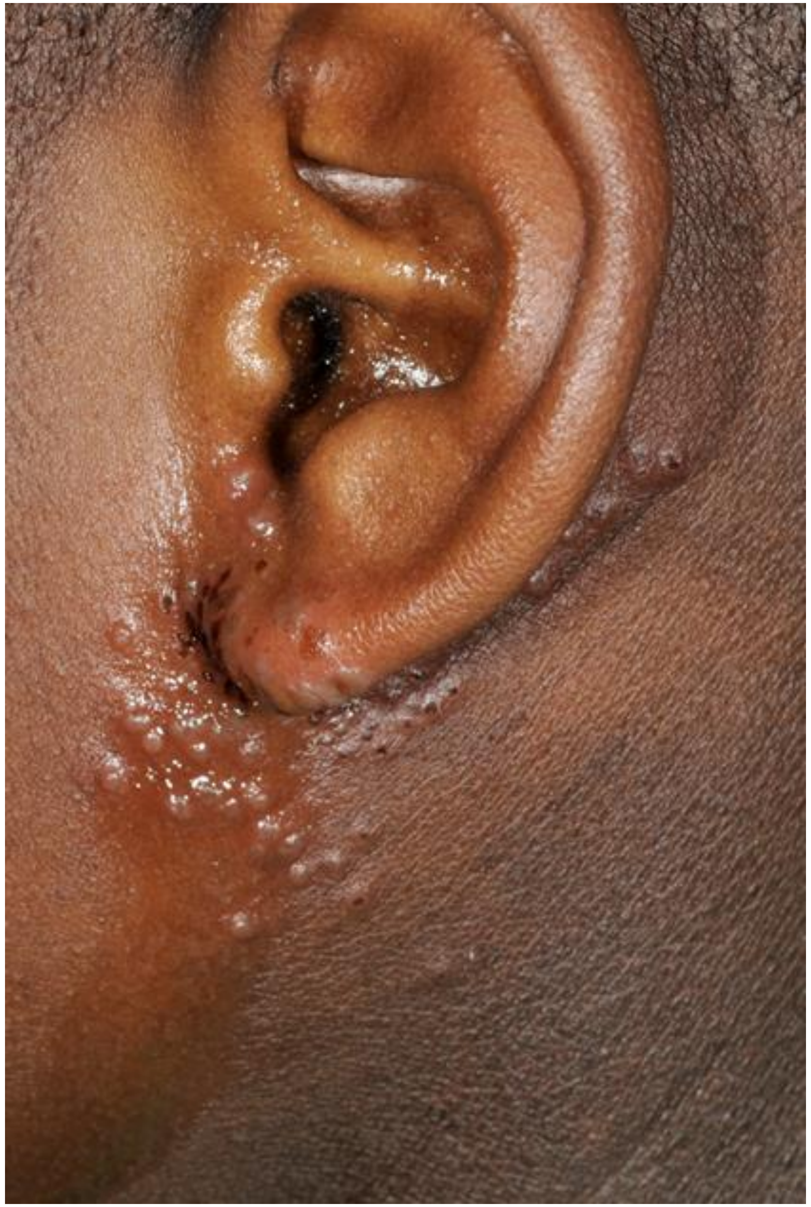

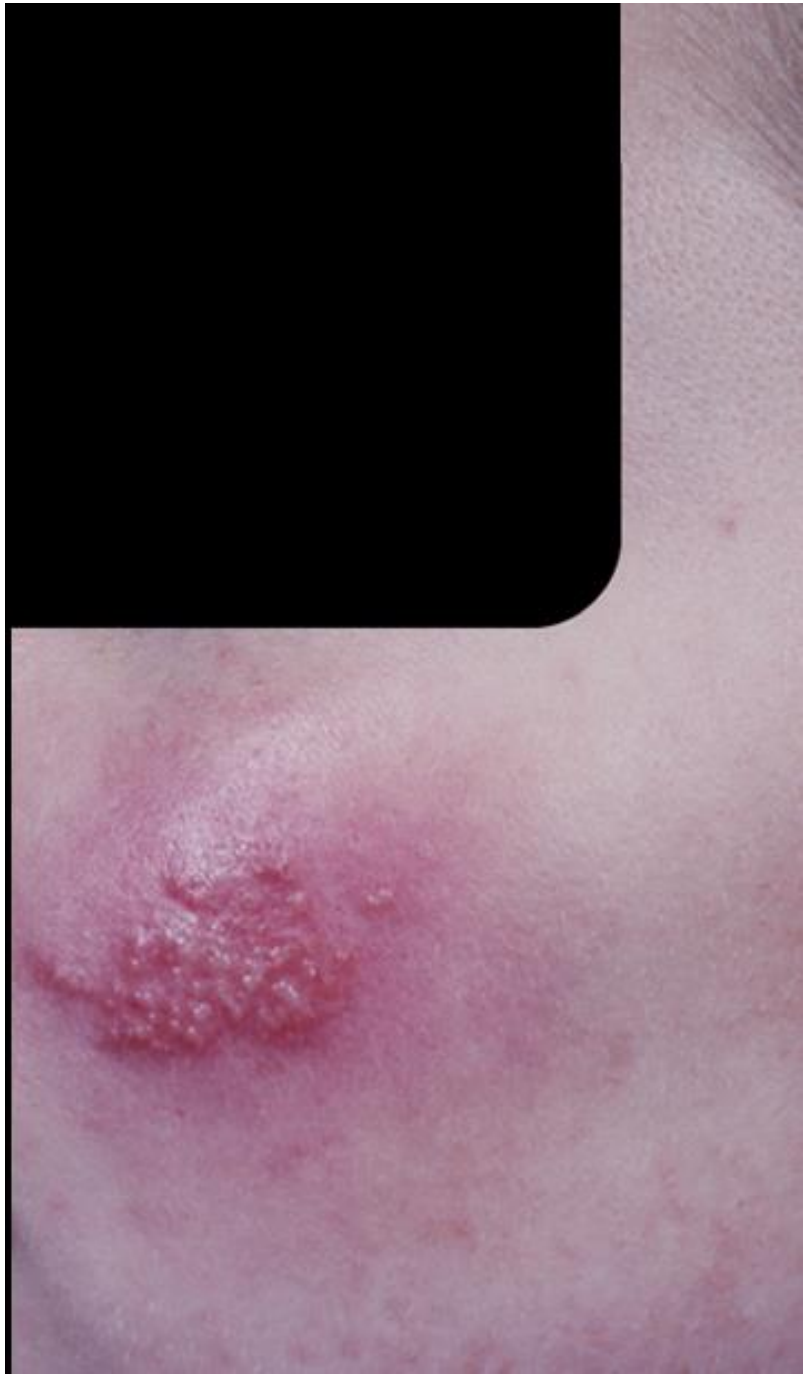

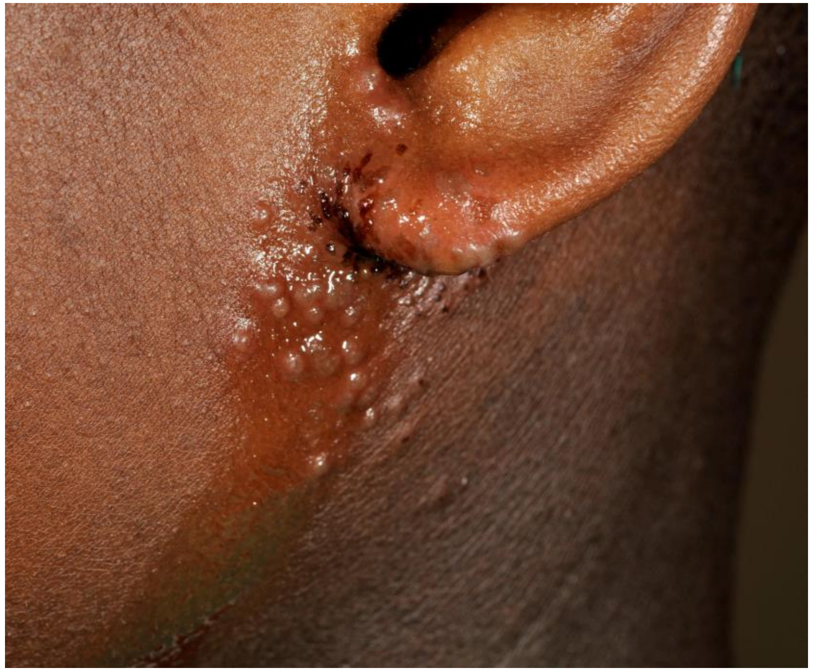

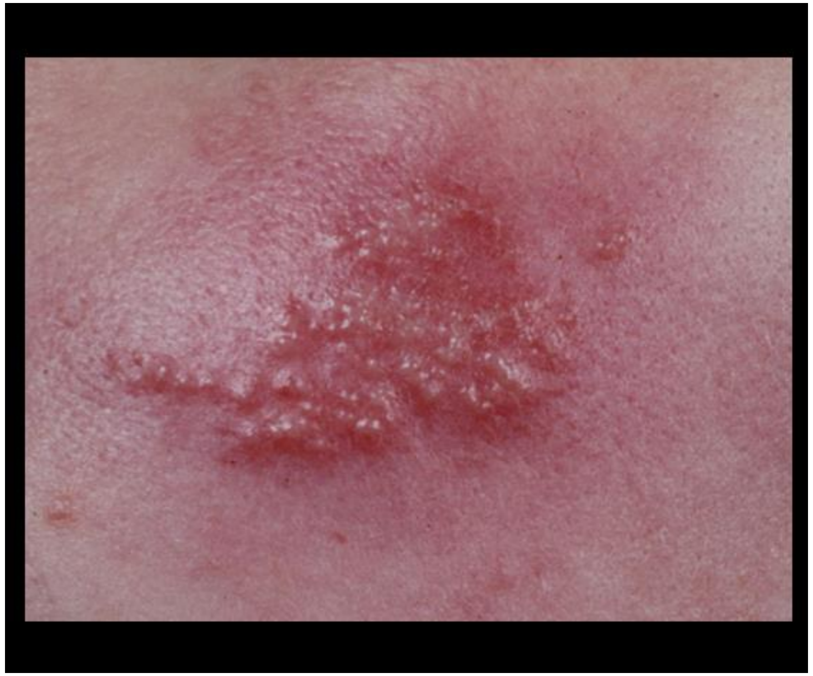

Correct answers: Herpes simplex

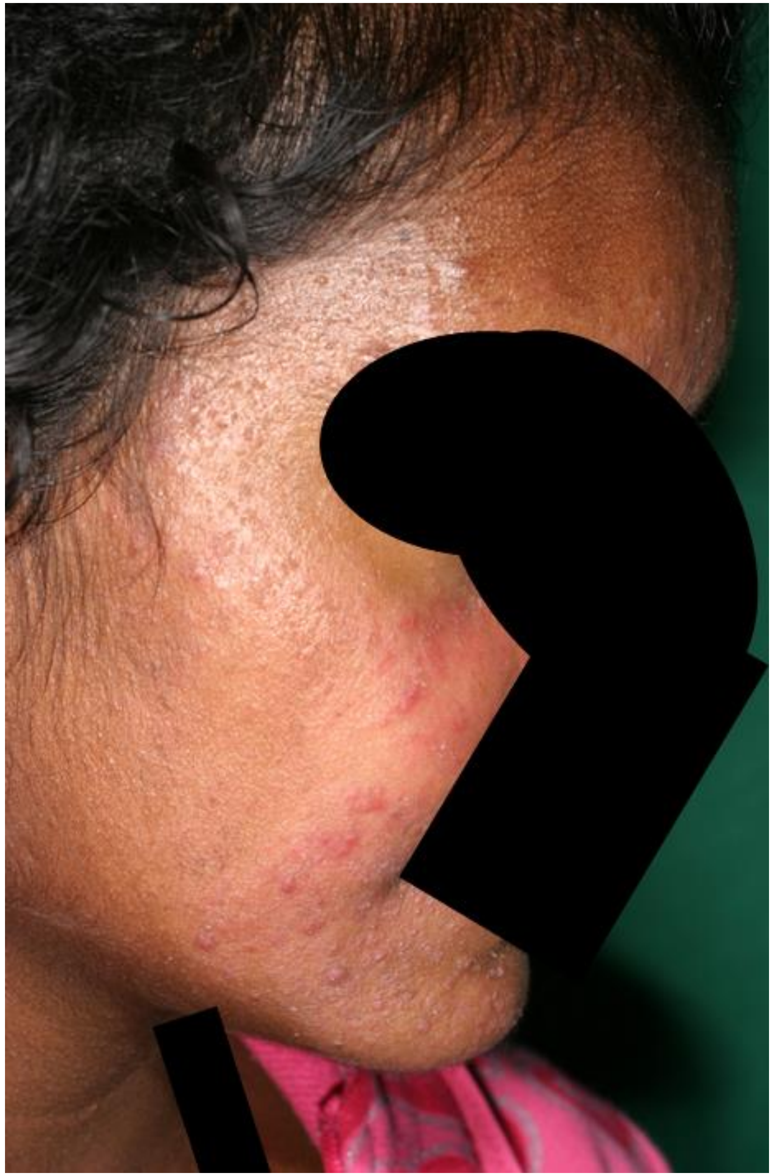

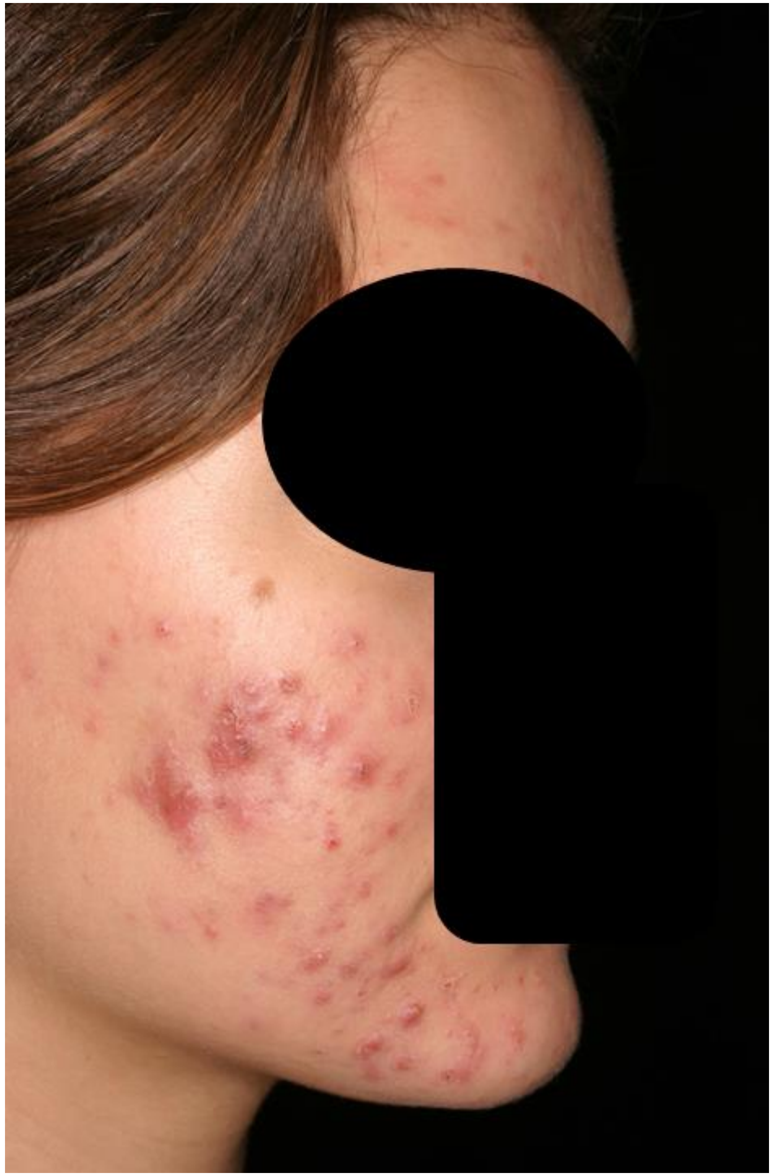

Correct answer: Acne
