# Supplementary material for: Comparison of visual diagnostic accuracy of dermatologists practicing in Germany in patients with light skin and skin of color
Source: Sci Rep. 2024 Apr 16;14:8740. doi: 10.1038/s41598-024-59426-4 (PMC11021442; doi:10.1038/s41598-024-59426-4)
Supplement: Supplementary file 2 — Supplementary Information. [file 41598_2024_59426_MOESM2_ESM.docx]

**Supplemental material:** Photographs included in the survey with the answers rated as correct for each case.
